# Tracking Musical Voices in Bach's *The Art of the Fugue*: Timbral Heterogeneity Differentially Affects Younger Normal-Hearing Listeners and Older Hearing-Aid Users

**DOI:** 10.3389/fpsyg.2021.608684

**Published:** 2021-04-14

**Authors:** Kai Siedenburg, Kirsten Goldmann, Steven van de Par

**Affiliations:** Department of Medical Physics and Acoustics and Cluster of Excellence Hearing4all, Carl von Ossietzky University of Oldenburg, Oldenburg, Germany

**Keywords:** music perception, hearing impairment, auditory scene analysis, voice leading, timbre

## Abstract

Auditory scene analysis is an elementary aspect of music perception, yet only little research has scrutinized auditory scene analysis under realistic musical conditions with diverse samples of listeners. This study probed the ability of younger normal-hearing listeners and older hearing-aid users in tracking individual musical voices or lines in JS Bach's *The Art of the Fugue*. Five-second excerpts with homogeneous or heterogenous instrumentation of 2–4 musical voices were presented from spatially separated loudspeakers and preceded by a short cue for signaling the target voice. Listeners tracked the cued voice and detected whether an amplitude modulation was imposed on the cued voice or a distractor voice. Results indicated superior performance of young normal-hearing listeners compared to older hearing-aid users. Performance was generally better in conditions with fewer voices. For young normal-hearing listeners, there was interaction between the number of voices and the instrumentation: performance degraded less drastically with an increase in the number of voices for timbrally heterogeneous mixtures compared to homogeneous mixtures. Older hearing-aid users generally showed smaller effects of the number of voices and instrumentation, but no interaction between the two factors. Moreover, tracking performance of older hearing aid users did not differ when these participants did or did not wear hearing aids. These results shed light on the role of timbral differentiation in musical scene analysis and suggest reduced musical scene analysis abilities of older hearing-impaired listeners in a realistic musical scenario.

## Introduction

In a process called auditory scene analysis (Bregman, 1990) the auditory system organizes sound mixtures into auditory events and streams. In the case of polyphonic music, this allows listeners to track distinct musical voices or follow a melody in the midst of an accompaniment. The notion of *voice* is used here in the music-theoretical sense of an independent musical line, often (but not necessarily) played by a single musical instrument or singing voice. Music psychology has long acknowledged the fundamental importance of auditory scene analysis in shaping music perception (McAdams and Bregman, 1979). Yet relatively little research has tested auditory scene analysis abilities under realistic musical conditions. Coffey et al. (2019) presented a music-in-noise task that had listeners hear out musical target melodies and rhythms from a masker signal consisting of four unrelated polyphonic music pieces artificially mixed together, but the ecological validity of this approach remains constrained. In addition, there is a scarcity of scene analysis research that reaches beyond young normal-hearing test participants. In this study, we attempted to address this void by comparing normal-hearing listeners' and hearing-aid users' ability of tracking voices in polyphonic excerpts from the music of JS Bach.

The compositional regularities underlying polyphonic music from the Baroque period are commonly referred to as *voice-leading rules*. These rules traditionally play an important role in Western music pedagogy, providing guidelines on how to construct polyphonic musical mixtures (also called *textures*) in which musical voices or lines move independently. The perceptual functions of voice leading have been portrayed by the work of Huron (2001, 2016). He described that “*the goal of voice leading is to facilitate the listener's mental construction of coherent auditory scenes when listening to music”* (p. 88). He also described the implicit pleasures derived from parsing a musical scene as being driven by the multiplicity of concurrent sound sources (i.e., musical instruments). Huron (2001, 2016) delineated how the traditional rules of voice leading can be derived from perceptual principles based on knowledge about auditory scene analysis. Two of these perceptual principles, as described in the following, are of particular concern for the present study because they relate to the number of concurrently active voices and the timbral heterogeneity of the mixture.

According to Huron (2016), the *limited density principle* states that the tracking of voices becomes difficult when the number of concurrently active voices exceeds three. Empirical studies using numerosity-judgment tasks that have participants judge the number of presently active voices in musical mixtures have corroborated this idea. In experiments using polyphonic excerpts from the music of JS Bach, Huron (1989) observed that errors in both numerosity judgments and the recognition of single-voice entries sharply increased from around ten percent for three-voice mixtures to around 50 percent for four-voice mixtures.

The *timbral differentiation principle* states that timbrally heterogeneous mixtures are easier to segregate compared to homogeneous mixtures. The empirical literature supporting this hypothesis is scarce, though. The so-called *Wessel illusion* (Wessel, 1979) shows that timbre cues can override pitch cues in auditory stream segregation for an isochronous sequences of artificially generated sounds. Bey and McAdams (2003) showed that timbre dissimilarity facilitates streaming in recognizing melodies within random-pitch distractor note sequences, also see McAdams (2019) and Marozeau et al. (2013). It is important to note that to the best of our knowledge timbre-based effects have not been empirically tested with music composed by reputed composers. Importantly, data on the limited density principle has only been collected for timbrally homogeneous mixtures (Huron, 1989, 2016).

Facing the fact that basic audiological conditions and age differ considerably across listeners, the above considerations gain yet another dimension. If one assumes that polyphonic music tends to *play with*, not against, the principles of auditory scene analysis in order to be apprehensible, a pertinent question becomes whether potentially hearing-impaired and older listeners can still participate in this sort of *play*. In fact, despite decades of substantial work on hearing impairment and speech, research on music perception has only started to address the effects of mild to moderate forms of hearing impairment on music listening (Madsen and Moore, 2014; Kirchberger and Russo, 2015; Choi et al., 2018); for research on cochlear implant listeners, see McDermott (2011), Marozeau et al. (2013), and Marozeau and Lamping (2019). Regarding the role of age in auditory scene analysis, Zendel and Alain (2013) found that younger musicians showed a greater tendency to segregate concurrent sounds compared to younger musicians and older musicians and non-musicians. More generally, aging has been shown to be associated with a reduced ability in exploiting spectral fine structure cues (Moore, 2015). Recent research on the specific aspect of musical scene analysis has shown that compared to younger normal-hearing listeners, older listeners with moderate hearing impairment have drastically higher thresholds for hearing out a musical melody or instrument in a simple chordal accompaniment (Siedenburg et al., 2020). Whether this reduced ability in scene parsing generalizes toward ecological musical scenarios (i.e., real-world music) remains an open question. Furthermore, whether hearing-aids, usually optimized for speech and not for music, have a beneficial role in musical scene analysis tasks has not been addressed under realistic conditions.

For these reasons, the present study sought to test musical scene analysis in a sample of younger normal-hearing listeners and older hearing-aid users with short excerpts from JS Bach's *The Art of the Fugue*. We used an indirect task that probed listeners' ability to detect alterations in the sound signal of the target voice: After a short cue presenting the beginning notes of the target voice, listeners were presented with 2–4 voice excerpts and were asked to indicate whether there was an amplitude modulation (i.e., tremolo effect) imposed on the cued voice. Stimuli were operationalized as to test the limited density principle with 2, 3, and 4 concurrently active voices and timbral differentiation principles with homogeneous and heterogeneous instrumentation. We also tested potential benefits of hearing aids by repeating the experiment with and without hearing aids, and included an analogous condition for normal-hearing listeners via low-pass filtering. We hypothesized that our experiment would confirm both the limited density and timbral differentiation principles and that older hearing-aid users would have poorer performance compared to younger normal-hearing listeners. We also expected hearing-aid users to perform better with hearing aids compared to without hearing aids, because the audibility of high frequencies would be restored. Similarly, we expected normal-hearing listeners to perform better without low-pass filtering compared to with low-pass filtering.

## Method

### Participants

This study tested young normal-hearing participants (yNH) and older hearing-aid users (oHA). Due to the Covid-19 pandemic, there is smaller number of oHA participants in this study (and the testing of a sample of older normal-hearing participants could not be completed). All participants received monetary compensation for their time. Initially, 21 yNH and 14 oHA participants were invited to the laboratory. For participation in the main experiment, participants were required to complete a training session (see section Procedure) with at least 80% of correct responses. Two yNH and three oHA participants did not pass the training and were hence not considered in the main experiment. In the 12 conditions of the main experiment, three oHA participants achieved levels of performance that were indistinguishable from chance, as indicated by right-tailed t-tests of each participant's distribution of scores against chance, *t*_(11)_ < 0.77, *p* > 0.22. These three oHA participants were excluded from any further data analysis. All subsequent analyses consider the remaining 19 yNH and 8 oHA participants.

**Figure 1:**
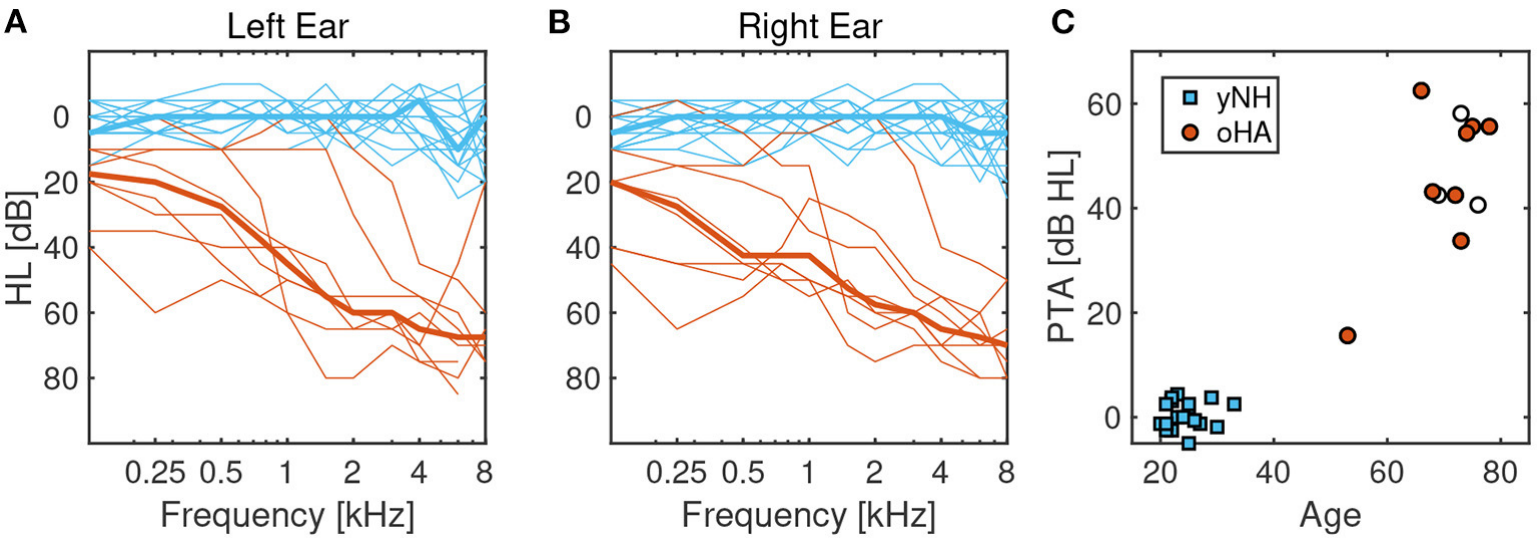
**(A)** Left-ear pure tone audiometric thresholds of younger normal-hearing (yNH) participants in blue and older hearing-aid users (oHA) in red. Thin lines correspond to individual participants, thick lines correspond to the mean across participants. **(B)** Right-ear pure tone audiometric thresholds. **(C)** Scatterplot of age and pure tone average thresholds (PTA) at 0.25, 1, 2, and 4 kHz. White circle-shaped symbols correspond to three oHA listeners who were removed from the analysis due to performance levels indistinguishable from chance.

The 19 yNH participants (six female, 12 male, one diverse) had a mean age of 25 years (*STD* = 3.5) and mean pure-tone thresholds (PTA, measured at 0.5, 1, 2, and 4 kHz) of 0.4 dB HL (*STD* = 2.6). The 8 oHA participants (4 female, 4 male) had a mean age of 70 years (*STD* = 7.8) and mean PTAs of 45.4 dB HL (*STD* = 15.2), see Figure 1 for a visualization. Hearing thresholds of most oHA participants were symmetric with the mean absolute difference between thresholds from the left and right ear being below 5 dB for six participants; there was one participant with asymmetries of 12 dB and another participant with asymmetries of 15 dB. All oHA participants used bilateral behind-the-ear digital hearing aids from major brands (Phonak, Oticon, Starkey, Unitron) that had been professionally fitted. All oHA participants were instructed to use the hearing-aid settings they would normally use when listening to or making music; two oHA participants indicated that they would use a music program for these purposes.

We measured musical training using the corresponding self-report inventory of the Goldsmiths Musical Sophistication Index Müllensiefen et al. (2014). It includes the following items (with weights of the cumulative index provided in brackets): Years of regular daily practice (1.57), no. of instruments played (0.82), having been complimented on performances [0: never, 1: always] (0.72), no. of hours practiced in period of peak interest (0.71), years of music theory training (1.43), years of instrument training (1.67), considers self musician [0: fully disagree, 1: fully agree] (0.90). The index yielded mean scores of 47.2 (*STD* = 16.8, median = 50.4, range: 19 − 94) for yNH participants and 45.7 (*STD* = 28.1, median = 45.5, range: 0−87) for oNH participants. A Wilcoxon rank sum test did not indicate different medians of these musical training scores the two groups of participants (*z* = 0.07, *p* = 0.94).

### Stimuli and Apparatus

Stimuli were chosen from an eminent piece of counterpunctual writing, namely JS Bach's *The Art of the Fugue* (BWV 1080, *Die Kunst der Fugue*). The work comprises 14 fugues and 4 canons with up to four voices. Notably, the instrumentation was not specified by the composer and thus it has been performed with a variety of different instrumentations. Using a MIDI-rendition of this piece, four-voice excerpts of around 5 s duration were extracted. Care was taken to avoid excerpts with the following features: termination of voices, parallel motion between voices, overlapping pitches between neighboring voices, or phrase boundaries. Overall, 72 excerpts were selected, from which 24 excerpts were presented as full four-voice mixture, 24 excerpts were presented as three-voice mixture by removing the bass voice, and 24 excerpts were presented as two-voice mixture by removing the bass and soprano voices. This approach hence employed the different number of voices as an operationalization of musical scene density, independent of potentially confounding musical properties or compositional intentions.

MIDI files were rendered using the Vienna Symphonic Library (www.vsl.co.at), based on high-quality recordings of orchestral instruments. In a homogeneous instrumentation condition, the soprano, alto, tenor, and bass voices were played in a string-quartet setting with violin 1, violin 2, viola, and cello, respectively. In a heterogeneous instrumentation condition, these voices were rendered using instruments from four separate instrument categories: flute (woodwinds), violin (strings), French horn (brass), and bassoon (double-reeds).

**Figure 2:**
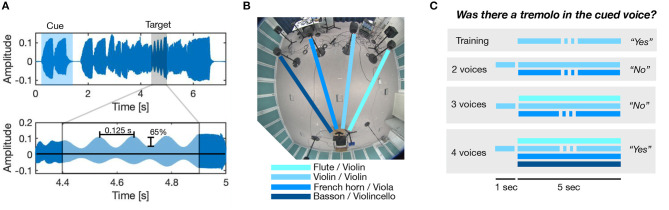
**(A)** Illustration of amplitude modulation (AM) manipulation. An excerpt of 0.5 s in the middle portion of the sounds was processed with AM with a modulation depth of 65% and a modulation rate of 8 Hz (i.e., modulation period of 0.125 s). **(B)** Lab setup for experiment. An experimental participant was seated in front of four loudspeakers in an acoustics lab. For the heterogeneous instrumentation, voices were played by the flute, violin, French horn, and bassoon; for the homogeneous instrumentation, voices were played by the instruments of a string quartet. **(C)** Visualization of the experimental task. Listeners were presented a cue, followed by a mixture, and they were asked to decide whether there was an amplitude modulation (AM) in the cued voice.

In every trial, an amplitude modulation (AM, “tremolo effect”) was applied for 0.5 s on the alto voice in one half of the trials and on the tenor voice in the other half of trials. See Figure 2A for an illustration. The AM had a sinusoidal shape and was generated with the MTremolo plugin (https://www.meldaproduction.com/MTremolo). In order to avoid salient “pop-out effects” of the modulated voice, which we had observed using regular sinusoidal AM (which both amplifies and attenuates the carrier signal), we chose to use a modulator that solely attenuates the carrier. The modulator signal *s*(*t*) can be described according to the following function, $s\left(t\right)=1-\frac{m}{2}\left(1+cos\left(2\pi f_{m}t+\pi \right)\right)$ where *m* is the modulation depth, *f*_*m*_ the modulation frequency, and *t* denotes time in seconds. Here, *f*_*m*_ = 8 Hz (AM-period: 125 ms) with a modulation depth of 65% (*m* = 0.65). The excerpts and positions of the AM manipulations were chosen such that the AM could be applied on only a single tone in order to avoid simultaneous pitch shifts in the manipulated voice; the onset of the AM was between 1 and 4 s after the onset of the mixture.

The oHA participants were tested with and without their oHA in separate sessions. To create an analogical test contrast for yNH participants, a filter with low-pass characteristic was used to process the stimuli, which served as a rudimentary way of simulating a hearing loss. This filter was created to attenuate high frequencies according to a standard profile of moderate high-frequency hearing loss that the group average of oHA users corresponded to (the N3 standard, see Bisgaard et al., 2010). Specifically, the filter featured an attenuation of –35 dB up until 0.75 kHz and from there up to 8 kHz had a slope of –10 dB/octave (i.e., implementing an attenuation of –70 dB at 8 kHz). These different acoustic situations for oHA users (with vs. without HA) and yNH listeners (without vs. with low-pass filtering) are henceforth referred to as acoustic conditions.

Participants were tested individually in an acoustic laboratory of the dimensions 6 x 7 x 2.7 m, see Figure 2B, which had a reverberation time T60 of 0.3 s. Participants were seated on a chair with a distance of 1 m from the rear wall with a computer monitor for presenting the experimental instructions in front of them (but close enough to the ground not to cause acoustic shadowing of the participant). Stimuli were presented using a MATLAB script on the test computer (Dell OptiPlex 5060) using an RME ADI-8 QS sound interface. Genelec 8030A active monitors were used as loudspeakers. All four loudspeakers were positioned with 4 m distance to the participant and were separated from each other by 1.1 m (angular separation of 15 degrees). Every instrument was presented from a fixed loudspeaker (that is, position) with an average level of 80 dB SPL as measured with a Norsonic Nor140 sound-level meter. The speaker position from which the specific instruments were played was fixed throughout the experiment. Participants communicated with the experimenter via an intercom system.

### Procedure

The procedure was approved by the ethics committee of the University of Oldenburg. The experiment was administered in two sessions on separate days. In the first session, participants were presented with 10 examples that illustrated how individual test stimuli sounded with and without the amplitude modulation, that was introduced to them less technically as a tremolo effect. Subsequently, participants were given sufficient time to clarify any question regarding the task or the stimuli. Participants then completed a training session of 40 trials that required participants to assess for excerpts containing only one voice wether there was a tremolo effect in the excerpt or not. In the training session, feedback about correct responses was provided after every trial. Participants who did not manage to achieve more than 80% correct responses in the training session were not allowed to complete the remainder of the experiment. Participants were then presented with 10 polyphonic example trials.

In the subsequent main experiment, listeners were presented a cue signal that comprised between one to four tones from either the alto or tenor voice and was of approximately 1 s duration (the precise duration depended on the specific phrase because notes were not cut off). The cue was followed by 0.5 s of silence and a 5 s mixture, comprising two, three, or four consecutive voices. Participants were asked to assess whether the AM manipulation (tremolo effect) was part of the cued voice or not. In half of the trials the AM manipulation was used on the cued voice, and in the other half it was used on the alternative voice. See Figure 2C for a visualization of the task. Participants did not receive feedback on correct responses during the main experiment.

The acoustic conditions (NH participants: original vs. low-pass filtered; oHA participants: with oHA vs. without HA) were presented block-wise as separate sessions on separate days and the order of presentation was counterbalanced across participants. The two other experimental factors of instrumentation and the number of voices were presented in fully randomized order. Sessions lasted around 45 min on average and contained a break after around 20 min. At the end of the first session, participants completed a questionnaire on demographic information and their background of musical training. After the end of the second session, participants were debriefed about the purpose of the experiment and their individual results.

### Data Analysis

We follow the current recommendation from the American Statistical Association (Wasserstein et al., 2019) by refraining from dichotomizing statistical significance based on thresholded probability values (*p* < 0.05) and rather describe the empirical results in quantitative terms. Square brackets indicate 95% confidence (i.e., compatibility) intervals for a given estimate.

Trial-level accuracy was analyzed using a generalized binomial mixed-effect model (West et al., 2007). All mixed effects analyses were conducted with the software R (3.5) using the packages lme4 (Bates et al., 2014). Our model included random intercepts for each participant and each item (i.e., stimulus). Marginal means and confidence intervals as provided in the text were estimated from the fitted models using the *emmeans* package (Lenth, 2018). All binary categorical predictors were sum-coded. The key analysis results are provided as part of Table 1 in Appendix A. The table includes *p*-values adjusted for multiple comparison within the linear model (Cramer et al., 2016), using the false discovery rate (Benjamini and Hochberg, 1995).

## Results

Considering the main results of all participants included in the analysis, the grand average of the group of yNH listeners was 0.79, with 95%-confidence-interval [0.75, 0.83], which was more than 10 percentage points higher than the grand average of oHA users with 0.67 [0.59, 0.73] without any overlap of confidence intervals and a moderate contribution to the model, β = 0.33 [0.15, 0.53], *p* < 0.001 (all *p*-values here and in the following FDR-corrected).

**Figure 3:**
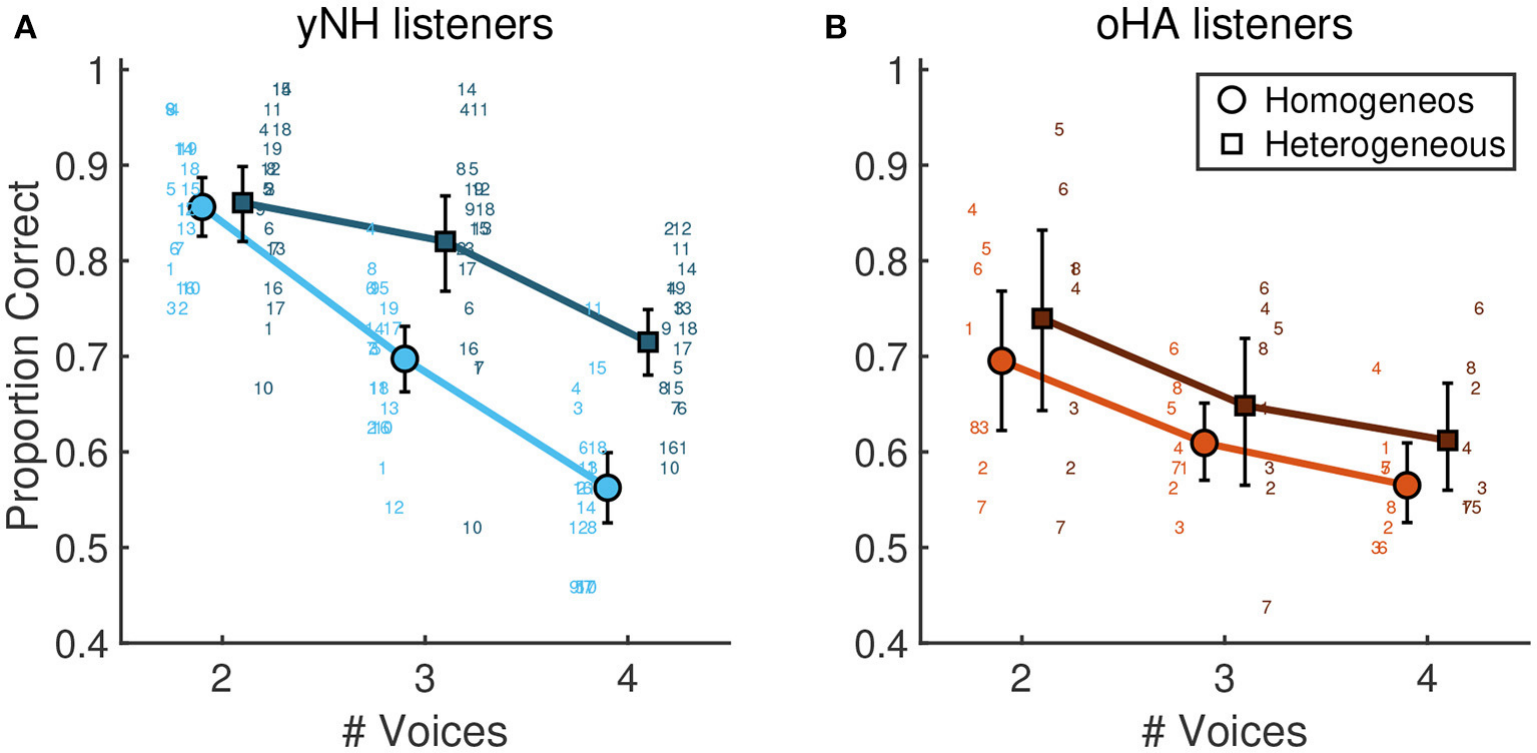
Mean proportion correct scores for different number of voices and instrumentations. **(A)** younger yNH participants (*n* = 19) and **(B)** older oHA listeners (*n* = 8). Errorbars correspond to 95% bootstrapped confidence intervals.

With increasing number of voices there was a strong decrease of scores for all participants, namely from average scores of 0.83 [0.77, 0.87] for two voices, 0.72 [0.65, 0.78] for three voices, and 0.63 [0.55, 0.71] for four voices. This was reflected in the model by a strong main effect of the number of voices, β = 0.54 [0.30, 0.76], *p* < 0.001 (for the factor contrasting 2 and 4 voices). Figure 3 depicts mean scores for yNH participants and oHA users in the training and test conditions, averaged across the two respective acoustic conditions of each of the two groups.

Average scores differed by around seven percentage points across instrumentations, with scores in the homogeneous instrumentation condition reaching 0.70 [0.64, 0.74] and in the heterogeneous instrumentation condition reaching 0.77 [0.72, 0.81] and β = −0.19 [−0.24, −0.14], *p* < 0.001. Importantly, yNH and oHA listeners appeared to exhibit a differential pattern of instrumentation effects: Scores of yNH listeners were by eight percentage points lower in the homogeneous condition (0.75 [0.70, 0.79]) compared to the heterogeneous condition (0.83 [0.79, 0.87]). On the contrary, oHA listeners showed a smaller improvement from the homogeneous (0.64 [0.56, 0.71]) to the heterogeneous condition (0.69 [0.61, 0.75]). This was reflected by the model in an interaction effect of group and instrumentation β = −0.076 [−0.13 − 0.02], *p* = 0.025, indicating that oHA were unable to benefit from timbral differentiation in the heterogeneous instrumentation in the same way as yNH listeners did.

As visible in Figure 3, there was an additional three-way interaction of group, the number of voices, and instrumentation, β = 0.131, [0.05, 0.21], *p* = 0.008. This three-way interaction indicated that yNH participants benefitted from heterogeneous instrumentation in mixtures with three and four voices, but not for two voices, whereas oHA participants did not show any differential effect of instrumentation as a function of the number of voices.

There were only negligible effects of acoustic condition (β = 0.025, *p* = 0.33) and no interactions with that factor: For yNH participants, average scores in the original vs. low-pass filtered conditions were strikingly similar (0.791 [0.75, 0.83] vs. 0.789 [0.74, 0.83]), and, surprisingly, the same hold for oHA participants with hearing aids compared to without hearing aids (0.68 [0.60, 0.74] vs. 0.66 [0.58, 0.72]). For that reason, the factor of acoustic condition was not considered any further in subsequent analyses.

**Figure 4:**
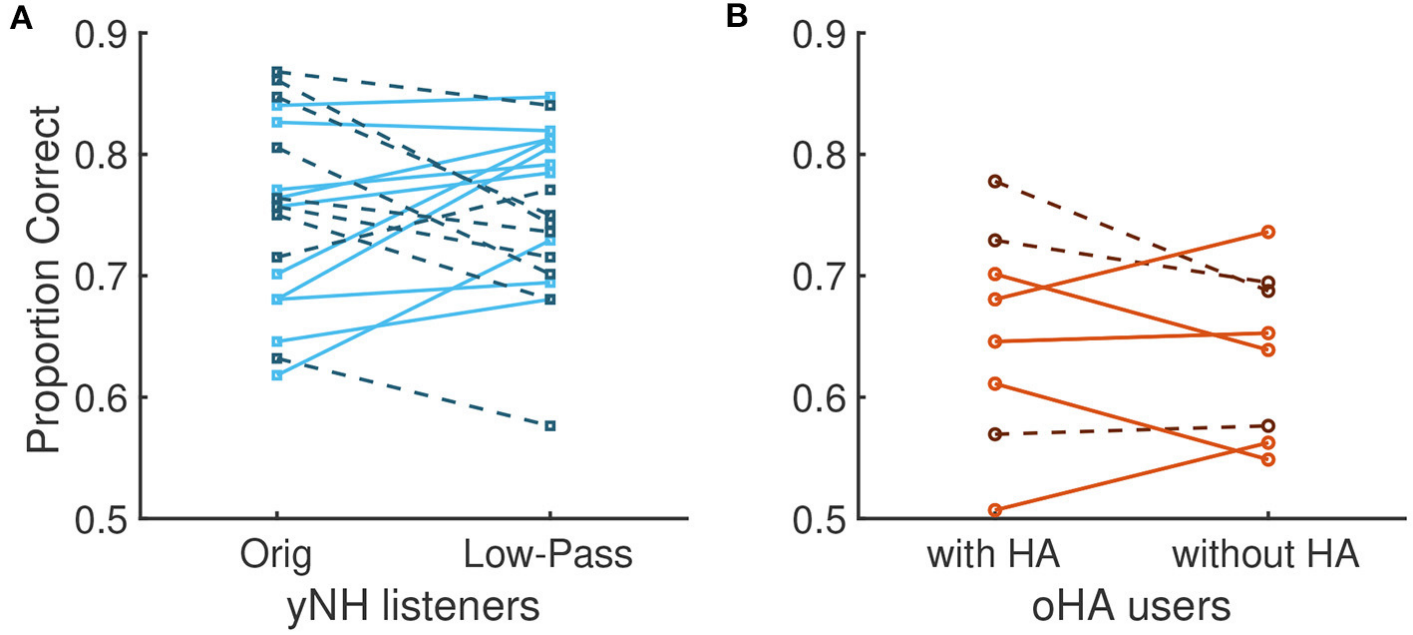
Proportion correct scores. **(A)** Data from individual participants for original and low-pass filtered conditions for yNH participants. Participants with solid lines first completed the original condition before completing the low-pass-filtered condition. Participants with dashed lines had the reverse order. **(B)** Data from oHA participants. Participants with solid lines first completed the test with oHA before passing the test without HA.

Figure 4 shows the individual results and averages for the two acoustic sessions for each of the two groups of participants. It also indicates the order by which participants conducted the first and second acoustic condition (solid lines correspond to the left-hand condition of the plot being presented first and the right-hand side being presented second; dashed lines correspond to the opposite). In contrast to the (non-existent) effect of acoustic condition, surprisingly, there was a strong effect of around 6 percentage points of the order of presentation: yNH listeners had a mean of 0.76 [0.71, 0.80] in the first session and a mean of 0.82 [0.78, 0.86] in the second session. However, oHA did not improve considerably across sessions with mean scores of 0.66 [0.58, 0.73] in the first session and 0.67 [0.60, 0, 74] in the second session. The model confirmed a moderate effect of order β = −0.11 [−0.17, −0.05], *p* = 0.001, as well as an interaction effect between group and order, β = −0.08 [−0.13, −0.01], *p* = 0.025. This interaction effect underlines that the improvement across sessions was particularly pronounced for the yNH participants.

Finally, adding the musical training index of participants to the model using the age predictor did not yield an effect of musical training, β = 0.08, *p* = 0.28 and only barely improved the model, χ^2^(27) = 3.98, *p* = 0.046 with fixed effects *R*^2^ = 0.102 compared to *R*^2^ = 0.101 for the dichotomous model without the musical training variable. Therefore, an index of musical training did not help to explain additional interindividual variability in the present sample of participants.

## Discussion

Musical scene analysis is at the heart of music perception, but relatively little research has addressed scene analysis in ecological scenarios. Present accounts of musical scene analysis suggest that *limited density* and *timbral differentiation* facilitate the parsing of musical scenes and the tracking of individual musical voices (Huron, 2001, 2016). However, to the best of our knowledge, the timbral differentiation principle has not been tested with real-world music and none of these principles has been tested with a task that allowed to objectively assess listeners ability to follow individual musical voices. Furthermore, experiments on musical scene analysis have traditionally focussed on young normal-hearing test participants. Performance estimates have hence not addressed potential impairments of auditory perception due to hearing impairment and aging.

In this study we addressed these questions by testing a sample of younger normal-hearing listeners and older hearing aid users. We used a tracking task that had listeners detect amplitude modulations in cued target voices in JS Bach's *The Art of the Fugue*. For younger normal-hearing listeners, we found an interaction of the factors of instrumentation and the number of voices: tracking performance was not different between homogeneous and heterogeneous instrumentations for two concurrently active voices, but there were substantial differences for three and four voices. For heterogeneous instrumentations of four concurrent voices, performance did not drop off sharply and was much higher than chance with 72% of correct responses, compared to around 56% of correct responses for the homogeneous condition. This finding indicates that the limited density principle with its strict cutoff at more than three concurrent voices may indeed serve as a guideline for timbrally homogeneous mixtures, but not necessarily for heterogeneous ones. It should further be noted that this study was designed such that the target was always presented in the inner voices, that is, the alto and tenor. If we had equally used outer voices as targets in the four-voice condition, this would have likely improved performance in these conditions (Trainor et al., 2014), potentially even more so challenging the limited density principle.

The performance of the small group of older yNH participants was qualitatively similar to that of younger yNH participants. For oHA participants, however, no interaction between instrumentation and the number of voices could be observed, but there was a small advantage for heterogeneous mixtures regardless of the number of voices. Conclusively, oHA users do not seem to profit as strongly from timbral differentiation compared to yNH listeners. The most striking difference between yNH and oHA listeners indeed occurs for four-voice mixtures, for which yNH listeners show much better performance for the heterogeneous instrumentation, but roughly equal performance to oHA listeners for the homogeneous instrumentation. This apparent inability to exploit useful cues for scene analysis is in line with the literature where hearing-impaired listeners have been shown to gain less from cues beneficial for stream segregation, such as spatial separation (Ernst et al., 2010) or differences in temporal fine structure (Moore, 2015).

The perhaps most unexpected outcome of this study was that the factor of acoustic condition did not have any effect on the results: here we contrasted the performance of oHA users with and without hearing aids tested in separate sessions. Similarly, there was no effect of low-pass filtering for yNH listeners, even though the differences between the two renderings were very salient. For yNH listeners, this may suggest that high-frequency information beyond 8 kHz may not be critical for scene parsing in ecological scenarios (although it certainly affects the perception of sound quality). However, one should note that this particular finding may be specific to the type of music used in the present experiment, that is, music without any percussive instruments and a relatively small number of instruments. We observed better tracking performance of yNH listeners in their second compared to the first experimental session. Generally, these findings may be interpreted as indicating that acoustical fidelity may not be absolutely necessary for successful scene analysis, but that cognitive schemata and implicit knowledge about musical structures can enhance musical scene analysis. This interpretation seems to be in line with studies on the rapid build-up of schemata in auditory scene analysis (Bey and McAdams, 2002; Woods and McDermott, 2018).

In the statistical analysis, adding the musical training index to the model did not improve the model fit. Note that previous studies of scene analysis found that musical training improved performance of participants in several scene analysis tasks (see e.g., Madsen et al., 2019; Siedenburg et al., 2020). In the present study, we did not undertake a dedicated comparison of groups of participants with and without musical training, and our musical training index mainly encoded variations in the degree of musical training. Together with the relatively small sample size, the variable may hence not have had sufficient power to relate differences of scene analysis to musical training.

We acknowledge several limitations of this study. Most importantly, the relatively small group of oHA participants leaves open the question whether the sample is representative for the highly diverse population of older hearing-impaired individuals. Furthermore, the lack of an age-matched control group does not allow us to draw any specific conclusions regarding the specific role of hearing impairment. Also, we did not control and test for varying cognitive abilities of participants, which may have well affected the present results. Although we trust the robustness of the present results regarding the differences between yNH and oHA listeners under realistic musical conditions, further research needs to disentangle the precise relation between the variables of age, cognitive abilities, PTA, musical training, as well potentially other important determinants of interindividual variability in musical scene analysis.

## Conclusion

We used a tracking task to assess the role the number of concurrent voices (i.e., density), timbral differentiation, and hearing aid usage in a realistic musical scenario using the music of JS Bach. We observed a graded decline of tracking performance from two to four voices, which was much less pronounced for heterogeneous mixtures and normal-hearing listeners. That is, timbral differentiation indeed appears to partially compensate for an increase of overall density in a musical scene. Notably, this compensation effect was not observed for older hearing-aid users, who showed overall poorer performance compared to younger normal-hearing listeners. Moreover, the performance of older hearing-aid users did not differ between conditions in which these participants did or did not wear hearing aids. Overall, these results may contribute to an increased awareness of the effects of hearing impairment on music perception and the present lack of adequate assistive hearing technology for music.

## Data Availability Statement

The raw data supporting the conclusions of this article will be made available by the authors, without undue reservation.

## Ethics Statement

The studies involving human participants were reviewed and approved by the Kommission für Forschungsfolgenabschätzung und Ethik of the University of Oldenburg. The participants provided their written informed consent to participate in this study.

## Author Contributions

KS, KG, and SP designed the research and revised the manuscript. KG collected the data. KS and KG analyzed the results. KS wrote the first draft of the manuscript. All authors contributed to the article and approved the submitted version.

## Conflict of Interest

The authors declare that the research was conducted in the absence of any commercial or financial relationships that could be construed as a potential conflict of interest.

## Appendix

**Table A1:** Generalized linear mixed model estimates with random effects: Fixed effects (marginal) *R*^2^ = 0.101; fixed and random effects (conditional) *R*^2^ = 0.25.

	**β**	**CI low**	**CI high**	**z val**	**p val**	**p adj**
intercept	1.013	0.812	1.247	8.7	<0.001	<0.001
group	0.326	0.153	0.528	4.1	<0.001	<0.001
voices24	0.541	0.303	0.755	4.2	<0.001	<0.001
voices34	–0.067	–0.249	0.217	–0.5	0.596	0.715
instr	–0.186	–0.248	–0.142	–6.3	<0.001	<0.001
order	–0.110	–0.169	–0.047	–3.8	<0.001	0.001
group:voices24	0.159	0.055	0.253	3.7	<0.001	0.001
group:voices34	0.033	–0.050	0.102	0.8	0.414	0.580
group:instr	–0.076	–0.129	—0.021	-2.6	0.009	0.025
voices24:instr	0.119	0.011	0.208	2.8	0.006	0.017
voices34:instr	–0.050	–0.140	0.047	–1.2	0.221	0.354
group:order	–0.075	–0.129	–0.006	–2.6	0.010	0.025
voices24:order	–0.034	–0.125	0.039	–0.8	0.428	0.580
voices34:order	–0.060	–0.137	0.033	–1.5	0.137	0.245
instr:order	0.043	–0.018	0.107	1.5	0.143	0.245
group:voices24:instr	0.131	0.048	0.213	3.0	0.002	0.008
group:voices34:instr	-0.066	–0.143	0.033	–1.6	0.105	0.218
group:voices24:order	–0.034	-0.135	0.057	–0.8	0.435	0.580
group:voices34:order	0.010	–0.058	0.099	0.2	0.807	0.842
group:instr:order	–0.017	–0.075	0.031	–0.6	0.571	0.715
voices24:instr:order	–0.069	–0.144	0.014	–1.6	0.109	0.218
voices34:instr:order	0.013	–0.053	0.094	0.3	0.758	0.826
group:voices24:instr:order	–0.004	–0.107	0.085	–0.1	0.927	0.927
group:voices34:instr:order	0.015	–0.066	0.095	0.4	0.718	0.821

*The rightmost column lists p-values adjusted for multiple comparisons (false discovery rate method). The variable group corresponds to the difference between yNH participants and oHA users. The variables voices24 and voices34 correspond to the contrasts between 2 and 4 voices and 3 and 4 voices, respectively. The variable order corresponds to the session order (1st vs. 2nd)*.
